# Evaluating the diagnostic accuracy of clinical judgement and rapid tests for leptospirosis in the Philippines: implications for public health management

**DOI:** 10.1186/s40249-026-01413-0

**Published:** 2026-01-22

**Authors:** Benjie Clemente, Peyman Ghoraishizadeh, Kween Saimuang, Sukhonta Limsampan, Patcharapan Suwannin, Edilberto Manahan, Kulachart Jangpatarapongsa

**Affiliations:** 1 Department of Medical Technology, Faculty of Pharmacy, University of Santo Tomas, España Boulevard, Sampaloc, Manila, 1008 Metro Manila the Philippines; 2 The Graduate School, University of Santo Tomas, España Boulevard, Sampaloc, Manila, 1008 Metro Manila the Philippines; 3 Center for Research Innovation and Biomedical Informatics, Faculty of Medical Technology, Mahidol University, 999 Phutthamonthon 4 Road, Salaya, Phutthamonthon, Nakhon Pathom 73170 Thailand; 4 School of Health Sciences, University of Santo Tomas, General Santos City, 1008 South Cotabato the Philippines

**Keywords:** Leptospirosis, Leptospira, Clinical diagnosis, Rapid testing, Sensitivity and specificity

## Abstract

**Background:**

Leptospirosis is endemic in the Philippines; however, its diagnosis remains challenging because of the lack of rapid and accurate diagnostic tools for detecting infection. Physicians must therefore resort to diagnosing leptospirosis through their clinical judgement, and this often results in under- or overestimation of cases. This study aimed to assess and compare the diagnostic accuracy of physicians’ clinical judgement and commercially available rapid test kits for leptospirosis against reference methods such as the microscopic agglutination test (MAT) and real-time polymerase chain reaction (qPCR) in the Philippines.

**Methods:**

A total of 127 serum samples were collected from patients suspected to have leptospirosis at three hospitals in the Philippines from August to December 2024. Rapid test kit results and final diagnoses were retrieved from the patients’ charts. MAT was performed on all the samples as a confirmatory method. Moreover, qPCR was performed on 30 randomly selected samples to increase the sensitivity of the reference standard. Sensitivity, specificity, positive and negative predictive values, and 95% confidence intervals were computed to determine the accuracy of both clinical judgement and rapid tests.

**Results:**

Among the 75 MAT-confirmed leptospirosis cases, approximately 24.0% were misdiagnosed as other febrile illnesses, such as dengue and typhoid fever, on the basis of clinical judgement, whereas 67.3% of the 52 MAT-negative patients were falsely diagnosed with leptospirosis. Overall, clinical judgement demonstrated high sensitivity (76.0%) but low specificity (33.7%), indicating possible overdiagnosis. The rapid test kits used in the laboratory exhibited significantly lower sensitivity (42.7%) but higher specificity (82.7%), suggesting a high probability of false-negative results. When qPCR was used in conjunction with these methods, relatively similar results were obtained.

**Conclusions:**

These findings highlight the diagnostic limitations in detecting leptospirosis in the Philippines, where laboratory testing options remain limited and inaccurate, resulting in physicians often relying on their clinical judgement. Misdiagnosis, whether through clinical judgement or rapid testing, could lead to inappropriate patient management, increased morbidity, and underestimation of leptospirosis incidence.

**Graphical Abstract:**

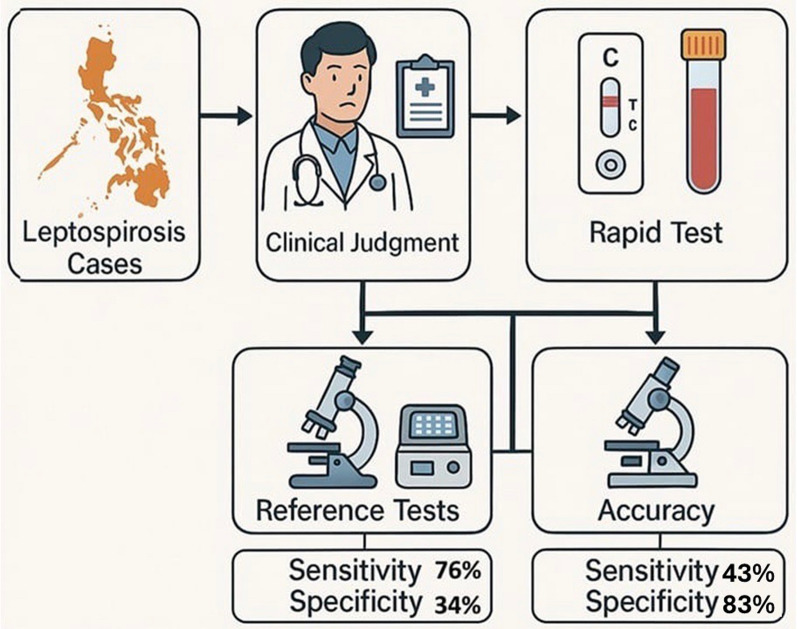

**Supplementary Information:**

The online version contains supplementary material available at 10.1186/s40249-026-01413-0.

## Background

Leptospirosis is a neglected zoonotic infectious disease that was first identified by Adolf Weil in 1886 [1]. Leptospirosis is caused by pathogenic species of the genus *Leptospira* and is considered a major threat to public health because of its widespread occurrence, particularly in developing countries with inadequate sanitation [2–6]. Infection of humans may occur via direct contact or host-to-host contact or indirectly through contact with soil or water contaminated with urine of infected mammals [7, 8]. Leptospirosis is known for its protean manifestations, which range from an absence of symptoms to patients with severe disease and the involvement of multiple organs [9, 10]. Owing to the nonspecific symptoms of leptospirosis, it can mimic other febrile infections, such as dengue, malaria, and flu, making diagnosis challenging [4, 11–14]. Failure to accurately diagnose leptospirosis may result in improper patient management, increased hospitalisation costs, and other adverse outcomes [15].

According to the World Health Organization, approximately one million individuals per year are infected by leptospirosis worldwide, and approximately 10% of these individuals succumb to the infection [16]. However, despite these numbers, the global disease burden of leptospirosis is underestimated because of limited prospective surveillance [17, 18]. Underestimation of leptospirosis burden has been reported in several countries [17, 19] and is rooted primarily in the lack of availability of diagnostic tests that detect the infection accurately [20–22]. In the Philippines, leptospirosis has been recorded throughout the year, but its incidence increases significantly during the rainy season. On the basis of a report from the Philippine Integrated Disease Surveillance and Response, Epidemiology Bureau, Department of Health, an average of approximately 3000 cases annually was reported from 2010 to 2022, with a case fatality rate of approximately 9% [23]. However, despite the consistent presence of leptospirosis in the Philippines, the disease remains underrecognised and underreported because of the diagnostic challenges faced in that country. Laboratory tests such as bacterial culture, the microscopic agglutination test (MAT), and polymerase chain reaction (PCR) testing, all of which offer high diagnostic accuracy, are available, but they are not readily accessible, especially in remote areas, and are time-consuming and expensive [24, 25]. Although other methods, such as loop-mediated isothermal amplification and biosensors, are increasing in popularity for the detection of leptospirosis, they are not yet widely used, and further studies are required to establish their diagnostic accuracy [26–32]. Commercially available serologic tests offer simplicity and rapidity in testing, but they often lack sensitivity and specificity for the detection of leptospirosis, especially when used during the acute stage of infection [33]. This underestimation of the morbidity and mortality due to leptospirosis contributes significantly to its neglected disease status and perpetuates a poor understanding of its epidemiology [5, 11].

Accurate and early diagnosis of leptospirosis is crucial for providing timely management and preventing the spread of the disease. However, in accordance with the guidelines set by the Department of Health (DOH) for leptospirosis in hospitals, confirmatory laboratory tests are not necessary for diagnosing leptospirosis infection. Owing to the low diagnostic accuracy and long turnaround times associated with laboratory tests, physicians may initiate treatment based on the patient’s clinical presentation. An individual is suspected of having leptospirosis if he or she (a) has a fever for at least two days; (b) exhibits two or more symptoms, such as calf discomfort, conjunctival suffusion, chills, abdominal pain, headache, jaundice, myalgia, or oliguria; and (c) has a high risk of infection on the basis of his or her clinical history. Clinical diagnosis is considered the most effective method of avoiding the occurrence of serious illness and mortality, especially in cases that involve epidemiologic risk factors and a high index of suspicion [34]. This limitation may lead to misdiagnosis, as leptospirosis infection, as noted above, presents with variable signs and symptoms. Thus, this study aimed to evaluate the diagnostic accuracy of clinical judgement as well as that of the rapid test kits used by our partner hospitals for leptospirosis detection.

## Methodology

### Patients and samples

Prior to the start of this study, ethics clearance was obtained from the Research Ethics Review Committee of Cagayan Valley Medical Center (RERC-CVMC 2024-31-E). All patients admitted to three hospitals in three different provinces (La Union, Cagayan, and Misamis Occidental) who were clinically suspected of having leptospirosis infection at the time of admission according to the 2019 DOH Guidelines for hospitals were included in this study. Those guidelines state that leptospirosis should be suspected if the patient has been febrile for at least two days, presents two or more symptoms of leptospirosis (these include but are not limited to myalgia, tenderness of the calf, abdominal pain, chills, headache, conjunctival suffusion, oliguria, and jaundice), and has a risk factor for contracting leptospirosis, such as living in a flooded area, having waded in contaminated water or floods, having had contact with the body fluids of animals, or having ingested contaminated water [34].

Only anonymised residual serum samples from patients suspected of having leptospirosis were used in this study. No samples were collected from patients specifically for the purpose of this study; instead, a volume of each of the serum samples used for leptospirosis rapid testing was collected prior to sample disposal. However, because there are no available records regarding the timing of sample collection, it cannot be determined whether samples were collected during the acute stage or the convalescent stage of the infection. A total of 127 single samples were collected at the three study sites from August to December 2024. All patients were subjected to rapid leptospirosis testing in the institution’s laboratory, and the results were recorded. The rapid test kits used in the samples employed immunochromatography and detected both IgM and IgG antibodies (Basecheck™ Leptospira IgG/IgM Rapid Test Cassette, Acro Biotech, China; Acon® Leptospira IgG/IgM Rapid Test, Acon Laboratories, USA; Aria® Leptospira IgG/IgM Combo Rapid Test, CTK Biotech, USA). Each patient’s final diagnosis was recorded for analysis in this study. Patients who lacked serum samples from previous laboratory tests or whose charts contained no record of the final diagnosis charts were excluded from the study. To ensure anonymity and confidentiality, no information on the patients other than their final diagnoses and their rapid leptospirosis test results was acquired.

### Leptospirosis case definition

#### Microscopic agglutination test

As recommended in the 2019 edition of the DOH guidelines for leptospirosis in hospitals, a 21-panel MAT comprising pathogenic and nonpathogenic *Leptospira* serovars (Australis, Ictohemorrhagiae, Bratislava, Autumnalis, Bataviae, Losbanos, Canicola, Grippotyphosa, Ratnapura, Copenhageni, Andaman, Poi, Panama, Pomona, Pyrogenes, Ranaram, Manilae, Hardjo, Sejroe, Shermani, and Tarassovi) was performed on all samples. The MAT served as a confirmatory and gold standard test in this study [34]. In addition to the 21 serovars, the nonpathogenic *Leptospira* Patoc I strain, which is suggested by the DOH guidelines as an alternative to the 21-panel MAT [34], and eight additional serovars (Semaranga, Hurtsbridge, Djasiman, Mini, Rachmati, Celledoni, Saxkoebing, and Carlos) were also included to increase the sensitivity of the test, increasing the total panel to 30 serovars.

The serum samples were initially diluted 1∶100 by adding 1 µl of the serum to 99 µl of phosphate-buffered solution, and dilutions of 1∶200, 1∶400, 1∶800, and 1∶1600 were then prepared by twofold serial dilution of the initial dilution. Equal volumes of the prepared sample dilutions and live *Leptospira* serovar cell suspensions in Ellinghausen-McCullough-Johnson-Harris broth were mixed in 96-well microtiter plates, and the plates were incubated at 30 °C for 2 h in a microshaker covered with aluminium foil. Positive and negative controls were also prepared in separate wells. The mixtures were examined for agglutination at 40× magnification under a dark field microscope. The reciprocal of the highest serum dilution that displayed at least 50% agglutinated *Leptospira* serovars was considered the MAT titre. Samples with MAT titres of ≥ 400 are considered positive for leptospirosis.

#### Real-time PCR

Because single-sample MAT may not reliably detect acute infections, we randomly selected 30 samples for further analysis. Those 30 samples were subjected to real-time PCR (qPCR) to identify potential acute infections that may have been missed by MAT. This allowed more accurate detection of the presence of active pathogens and enhanced the overall diagnostic sensitivity of our study.

DNA was extracted from patient serum samples using the Presto™ Mini gDNA Bacteria Kit (Geneaid Biotech Ltd., New Taipei City, Taiwan). To determine the DNA concentration in the extracts, the absorption of each extracted DNA sample at 260 nm was measured using a NanoDrop™ One/Once Microvolume UV‒Vis Spectrophotometer (Thermo Fisher Scientific Inc., Massachusetts, USA). The presence of protein in each DNA extract was also tested by measuring the absorption of the extract at 280 nm. The samples were subjected to qPCR using a commercial BactoReal®Leptospira spp. (.LipL32) kit (Ingenetix GmbH, Vienna, Austria) to detect the *LipL32* gene according to the manufacturer’s instructions. The assay was performed in a CFX real-time PCR thermocycler (Bio-Rad Laboratories Inc., Hercules, CA, USA).

### Data analysis

The diagnostic accuracy of clinical judgement and of laboratory rapid test kits for detecting leptospirosis infection were determined. In particular, the sensitivity and specificity, as well as the positive predictive value (PPV) and the negative predictive value (NPV), were computed using the following formulas:

$$Sensitivity (\%)=\frac{true positives}{true positives+false negatives}*100$$

$$Specificity (\%)=\frac{true negatives}{true negative+false positives}*100$$

$$PPV (\%)=\frac{true positives}{true positives+false positives}*100$$

$$NPV (\%)=\frac{true negatives}{true negatives+false negatives}*100$$

A MedCalc® online diagnostic test calculator (https://www.medcalc.org) was used to compute the proportions and 95% confidence intervals of each parameter.

With respect to clinical judgement, “true positive” refers to confirmed positive samples and patients who were clinically diagnosed with leptospirosis, while “true negative” refers to confirmed negative samples and patients who were clinically diagnosed with conditions other than leptospirosis. On the other hand, “false-positive” refers to patients who were clinically diagnosed with leptospirosis but were confirmed not to have the disease, while patients who were not diagnosed with leptospirosis but were found to have the disease were indicated as false-negatives. Similarly, for evaluation using the institution’s rapid test kit for leptospirosis, “true positive” indicates a positive result in both the rapid test and the confirmatory test, whereas “true negative” refers to testing negative for both tests. A false-positive is indicated by a positive rapid test result that was confirmed to be negative, while false-negatives are samples that tested negative in the rapid test but were later confirmed to be positive for leptospirosis.

## Results

**Fig. 1:**
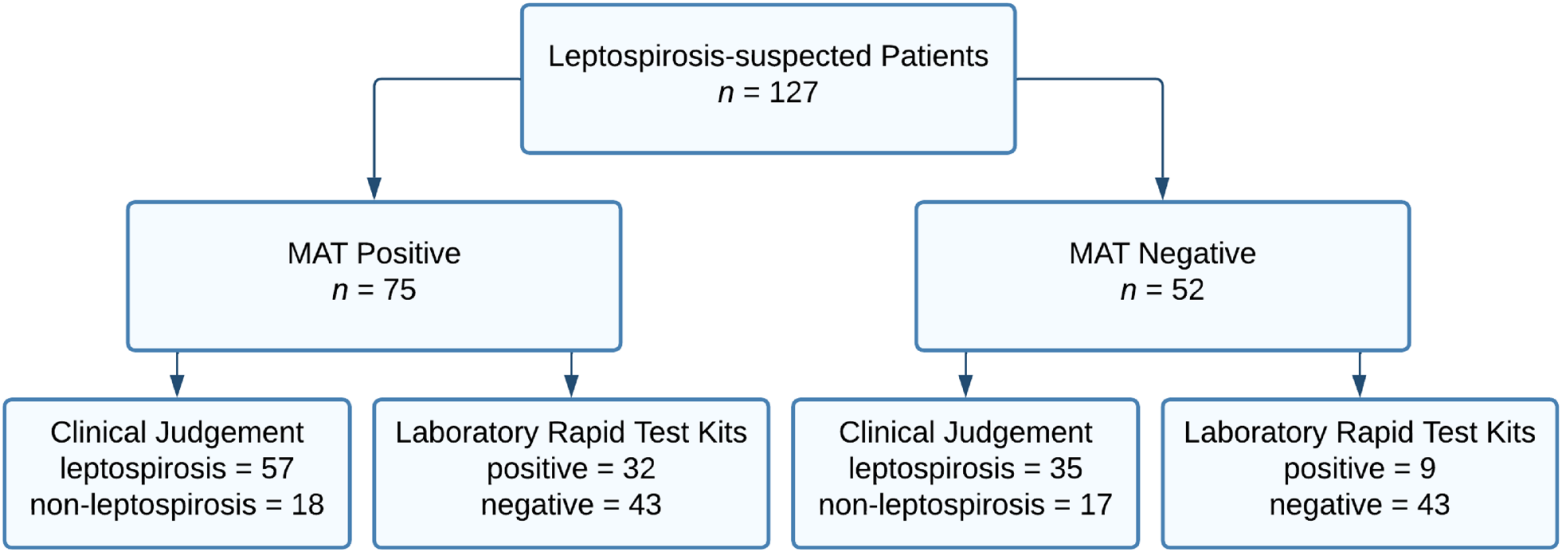
Flowchart of results of gold standard MAT, physicians’ clinical judgement, and laboratory rapid test kits for all leptospirosis-suspected samples. MAT: microscopic agglutination test

A total of 127 serum samples collected from leptospirosis-suspected patients admitted to our three partner hospitals were used in this study. The MAT results for all the samples, as well as the final diagnoses of the patients and the results of the rapid tests for leptospirosis conducted at their respective institutions, are shown in Fig. 1. A total of 75 patients (59.1%) were confirmed to be positive. Among these patients, 57 (76.0%) were clinically diagnosed with leptospirosis by physicians, while the remaining patients were misdiagnosed as having other diseases, such as unspecified viral infections, community-acquired pneumonia, dengue, or typhoid fever. With respect to the rapid test performed in the hospitals’ laboratories, only 42.7% of the 75 leptospirosis-confirmed samples tested positive. On the other hand, 35 (67.3%) of the 52 MAT-negative patients were clinically misjudged as having leptospirosis infection, and approximately 17.3% had false-positive results in the rapid leptospirosis test.

The overall diagnostic accuracies of clinical judgement and rapid test kits were computed to be 58.3% and 59.1%, respectively, indicating relatively similar performance of the two methods in correctly identifying leptospirosis cases (Table 1). However, the use of physicians’ clinical judgement was more sensitive (76.0%) in diagnosing leptospirosis than was rapid testing by the hospital’s laboratory (42.7%), indicating that the likelihood of identifying patients with leptospirosis is greater when the diagnosis is based on clinical judgement. Conversely, the rapid laboratory test was more specific for detecting leptospirosis; its specificity was 82.7%, compared with only 33.7% when the diagnosis was based on clinical judgement. These results indicate that laboratory rapid test kits are more effective at identifying true leptospirosis-negative patients than clinical judgment, thereby reducing the likelihood of overdiagnosis. In terms of the PPV and NPV, the laboratory rapid test provided higher predictive values (78.1% and 50.0%, respectively) than did clinical judgment, for which the values were 62.0% and 48.6%, respectively. These findings suggest that rapid laboratory testing is more likely than clinical judgement is to predict the presence or absence of leptospirosis in patients.

**Table 1:** Comparison of the diagnostic sensitivity and specificity of physicians’ clinical judgement and the institutions’ laboratory rapid test kits with the results of gold standard MAT alone (*n* = 127)

		MAT		Sensitivity (95%* CI*)	Specificity (95% *CI*)	PPV (95%* CI*)	NPV (95%* CI*)	Accuracy (95% *CI*)
		Positive	Negative					
Clinical judgement	Positive	57	35	76.0% (64.5%–85.1%)	33.7% (20.3%–47.1%)	62.0% (56.5%–67.2%)	48.6% (35.0%–62.3%)	58.3% (49.2%–67.0%)
	Negative	18	17					
Laboratory rapid test kits	Positive	32	9	42.7% (31.3%–54.6%)	82.7% (69.7%–91.8%)	78.1% (65.0%–87.2%)	50.0% (44.2%–55.8%)	59.1% (50.0%–67.7%)
	Negative	43	43					

*CI* confidence interval; *PPV* positive predictive value; *NPV* negative predictive value; *MAT* microscopic agglutination test

To overcome the limitations of single-sample MAT in detecting acute leptospirosis infection, we randomly selected 30 samples and performed qPCR to detect the presence of *Leptospira* spp. antigens in those samples. The, results are summarised in Table 2. As in the initial findings, physicians’ clinical judgement in diagnosing leptospirosis demonstrated higher sensitivity (82.6%) than did the use of rapid test kits (39.1%), indicating that clinical judgement is better at detecting true-positive leptospirosis cases and less likely to miss individuals with the disease. Clinical judgement showed better specificity (57.1%) when evaluated on the basis of the qPCR results than it did when evaluated on the basis of single-sample MAT alone; however, its specificity was still much lower than that of laboratory rapid test kits (71.4%), suggesting that it is more likely to generate greater false positives. With respect to the PPV, a significant change was observed for the use of clinical judgement (86.4%) compared with the previous result. Moreover, the NPV of the laboratory rapid test was also significantly lower at 26.3%. These results can be drawn from the smaller sample size used.

**Table 2:** Comparison of the diagnostic sensitivities and specificities of physicians’ clinical judgement and the institutions’ laboratory rapid test kits with the results of combined MAT and qPCR (*n* = 30)

		MAT + qPCR		Sensitivity (95%* CI*)	Specificity (95% *CI*)	PPV (95%* CI*)	NPV (95% *CI*)	Accuracy (95%* CI*)
		Positive	Negative					
Clinical judgement	Positive	19	3	82.6% (61.2%–95.1%)	57.1% (18.4%–90.1%)	86.4% (72.5%–93.8%)	50.0% (25.0%–75.0%)	76.7% (57.7%–90.1%)
	Negative	4	4					
Laboratory rapid test kits	Positive	9	2	39.1% (19.7%–61.5%)	71.4% (29.4%–96.3%)	81.8% (55.6%–94.2%)	26.3% (16.8%–38.8%)	46.7% (28.3%–65.7%)
	Negative	14	5					

*CI* confidence interval; *PPV* positive predictive value; *NPV* negative predictive value; *MAT* microscopic agglutination test; *qPCR* real-time polymerase chain reaction

Overall, the results obtained using the gold standard MAT alone and the MAT combined with qPCR were relatively similar, indicating consistency in how each reference method classified leptospirosis cases. When the results obtained using clinical judgement and rapid test kits were compared with the results obtained using these reference standards, clinical judgement demonstrated higher sensitivity (76.0%–82.6%), indicating that it was more likely to correctly identify true leptospirosis cases, but it recorded low to moderate specificity (33.7%–57.1%) and NPV (48.6%–50.0%), indicating that it may yield more false positives. In contrast, the rapid test kits showed lower sensitivity (39.1%–42.7%) and therefore missed more true cases, but it had higher specificity (71.4%–82.7%) and PPV (78.1%–81.8%), indicating that it is more reliable at ruling out non-leptospirosis cases.

## Discussion

Definitive diagnosis of leptospirosis, especially during its acute phase, is essential to guide physicians in providing appropriate treatment and patient management. Early diagnosis aids in preventing the development of detrimental complications, thereby possibly lowering mortality due to the infection. However, despite advancements in technology in the field of medicine and diagnostics, the Philippines continues to face challenges in the diagnosis of leptospirosis. Highly specific laboratory tests such as MAT, PCR, and bacterial culture are not routinely performed in the Philippines and are offered in only a limited number of laboratories nationwide. Most physicians in the country rarely request such tests because of difficulties in transporting samples and delays in obtaining results. Although rapid tests such as immunochromatographic test kits are commercially available in, they generally have low sensitivity and specificity for the rapid detection of leptospirosis [33], and some hospital-based laboratories do not offer such tests. This dilemma makes it necessary for physicians to make a diagnosis of leptospirosis on the basis of their clinical judgement of patients’ epidemiologic risks and index of suspicion [34].

This study investigated the under- and over-recognition of leptospirosis in the Philippines. A considerable number of suspected leptospirosis patients were confirmed using the gold standard MAT; most of these patients reacted positively to the serovars Shermani, Semaranga, and Tarasovi. While most of these cases were accurately identified as leptospirosis, a notable portion was incorrectly diagnosed as other febrile illnesses such as dengue, typhoid, and pneumonia. These findings are in agreement with those of other studies in which patients were treated for dengue infection and other infections but tested positive for leptospirosis [35–37]. On the other hand, more than half of the MAT-negative patients included in this study were misdiagnosed with leptospirosis. According to Gasem et al., overdiagnosis of leptospirosis is common in areas where infection is prevalent, especially when patients present with typical manifestations of leptospirosis such as jaundice and conjunctival suffusion [37]. Basing the diagnosis of leptospirosis solely on clinical judgement can be challenging, and it is highly prone to inaccuracies. In this study, although the use of clinical judgement presents high sensitivity, it lacks substantial specificity, leading to a high possibility that the incidence of leptospirosis in the Philippines is being underestimated. Underestimation of the incidence of leptospirosis has also been a problem in most developing countries because of the lack of definitive diagnostic tools for the detection of the disease [5, 38].

Several rapid laboratory tests for the detection of leptospirosis are currently available in the market. In the Philippines, approximately 20 such rapid test devices were registered in the Philippine Food and Drug Administration as of September 27, 2024 [39]. Most of these rapid tests use immunochromatography to detect antibodies in patients’ blood against *Leptospira* species*.* However, to our knowledge, no published literature has evaluated the diagnostic accuracy of these rapid test kits in the setting of the Philippines. In a systematic review conducted by Clemente et al. (2022), it was emphasised that the diagnostic performance of rapid test kits should be evaluated in the local setting before their use. Their review of the diagnostic accuracy of several commercially available immunochromatographic test kits revealed a wide range of sensitivities and specificities when they were used in different countries [33]. In this study, we also evaluated the diagnostic accuracy of the rapid test kits used by our partner hospitals to detect leptospirosis. Although each hospital uses a different brand of test kit, we assessed the diagnostic accuracy of the kits as a whole and did not evaluate the different brands individually. Nevertheless, our study revealed that the overall sensitivity of the rapid test kits used by our partner hospitals appears to be limited compared to the sensitivity of MAT, the reference standard. This low sensitivity increases the likelihood of false-negative results, meaning that many leptospirosis-infected individuals may be incorrectly classified as disease-free or misdiagnosed with other infections [40]. Although rapid test kits demonstrate high specificity, this alone cannot compensate for their poor case-finding capability, particularly when they are relied upon in early clinical decision-making where timely diagnosis is crucial. These findings are comparable to those of several studies that also revealed high specificity but low sensitivity of the test kits when evaluated in the local setting [41–45]. As a result, reliance on rapid test kits alone for the diagnosis of leptospirosis may contribute to substantial underdiagnosis in affected populations.

Given the low sensitivity of single-sample MAT for the diagnosis of leptospirosis, we supplemented our confirmatory testing with qPCR to overcome this limitation. This molecular approach increases the diagnostic accuracy of confirmatory testing, particularly during the early phase of infection when antibodies against the bacterium are not yet detectable [46]. A random subset of 30 of the total samples was subjected to qPCR, and relatively similar results were obtained compared with the single-sample MAT alone. We are reasonably confident that the sample selection was unbiased. This sample size represents the minimum required to ensure statistical validity of the study while still ensuring reliable estimates of diagnostic accuracy and precision. Although both MAT and qPCR are recommended by the DOH for confirmatory testing, the implementation of these methods remains limited to reference laboratories and is not routinely performed. Scaling up these tests in a way that allows their use in the diagnosis of leptospirosis in the Philippines presents a complex array of operational and economic challenges. One of the primary hindrances to the use of these tests is the high cost of establishing and maintaining the infrastructure that is necessary to conduct them. Furthermore, conducting the tests requires a high level of technical expertise and sophisticated equipment [47, 48]. Although MAT is referred to as the gold standard for the diagnosis of leptospirosis, it is not useful for patient management because of the long turnaround time needed to obtain results [46].

The results of the current study suggest that leptospirosis may be both under- and overreported in the Philippines because of a lack of availability of highly accurate laboratory tests for detecting the infection, resulting in physicians relying on their clinical judgement for diagnosis. These findings are similar to those obtained in other countries as well as in neighbouring Southeast Asian countries in which leptospirosis is also prevalent [22, 49–53]. While clinical judgement based on epidemiologic risks and an index of suspicion is valuable, it is insufficient when used alone. The findings of this study show that although clinical judgement tends to identify more true cases than the use of rapid test kits does, it performs poorly in identifying individuals without disease. These results reveal overdiagnosis, which can lead to the unwarranted administration of broad-spectrum antibiotics, thereby exacerbating antimicrobial resistance and diverting attention from differential diagnoses of dengue, malaria, or rickettsial infections. In contrast, the rapid test is better than clinical judgement at correctly excluding non-leptospirosis cases and provides a higher likelihood that a positive result truly reflects infection; however, it fails to detect a substantial number of actual cases, leading to underdiagnosis. This may result in delayed initiation of appropriate antimicrobial therapy and in increased risk of severe complications such as acute kidney injury, hepatic dysfunction, pulmonary haemorrhage, and death. This is particularly critical in regions in which the disease is endemic and in the context of environmental exposures such as flooding. Misdiagnosis also skews epidemiological data, hindering effective surveillance and resource allocation. Taken together, the results indicate that neither method alone is sufficiently reliable to provide accurate case detection or estimation of the disease burden. Therefore, there is a pressing need to develop and implement more sensitive and specific diagnostic modalities to enhance case detection, inform appropriate treatment, and guide public health interventions.

The results of this study suggest that future research should focus on developing and validating a standardised national diagnostic algorithm for leptospirosis diagnosis that integrates clinical, epidemiological, and laboratory data and thereby increases diagnostic accuracy. The evaluation of newer, more sensitive rapid test kits that are affordable, fast, and feasible for use in resource-limited areas in the country is vital. We also recommend assessing combinations of clinical criteria with biological markers such as inflammatory or renal function parameters to enhance case definitions and improve early case detection. Moreover, validation studies of commercially available rapid test kits in the local setting are highly warranted to ensure the accuracy and reliability of these test kits in the context of the Philippines. Epidemiological research exploring regional differences in *Leptospira* serovars, transmission patterns, and cocirculating febrile illnesses can further inform the development of a tiered, context-specific diagnostic framework. These efforts are crucial for supporting clinicians in accurately diagnosing and managing leptospirosis and will ultimately reduce the number of misdiagnoses and improve patient outcomes nationwide. Since only residual samples from suspected leptospirosis patients were used in this study, the timing of sample collection was not considered. Additionally, only a single sample from each patient was used. These limitations may have led to false-negative results for MAT, especially when the samples were taken during the early phase of infection when antibodies had not yet developed [1]. To address this limitation, we performed qPCR and obtained similar results. To validate and extend the current findings, larger-scale studies involving multiple centres and a more representative sample size are recommended to improve the external validity of the results. Coinfection with leptospirosis was not assessed in our study since only the results of the rapid test from the hospitals’ laboratories were recorded, and other information and the results of other laboratory tests were not obtained. Another limitation is that we did not perform other tests, such as bacterial culture, to confirm leptospirosis infection.

## Conclusions

Leptospirosis is considered an endemic infection in the Philippines, with cases and fatalities recorded throughout the year. However, definitive diagnosis of leptospirosis remains a substantial challenge in the country because of the limited availability of accurate and rapid laboratory tests and the reliance on physicians' clinical judgement. This study aimed to evaluate the diagnostic accuracy of human leptospirosis using clinical judgement and laboratory rapid test kits, with the MAT serving as the gold standard. The findings revealed that clinical judgement demonstrated higher sensitivity than laboratory rapid test kits did, indicating a better ability to detect true-positive cases and thus reducing the likelihood of misdiagnosis. However, the rapid test kits exhibited significantly better specificity, suggesting that they are more effective at correctly identifying individuals who do not have the disease. The laboratory rapid test kits showed higher positive and negative predictive values than did physicians’ clinical judgement, indicating greater reliability in both confirming and excluding leptospirosis cases based on the test results. These outcomes highlight the strengths and limitations of each diagnostic approach. Although rapid test kits offer speed, their low sensitivity limits their standalone diagnostic utility, leading to potential misdiagnosis. This study highlights the potential for both under- and over-recognition of leptospirosis and emphasises the need for improved diagnostic strategies. Improving diagnostic capabilities is crucial for effectively managing patients and providing timely treatment to reduce the burden of leptospirosis in the Philippines.

## Supplementary Information

Supplementary material 1.

## Data Availability

All the datasets analysed in this study are available and may be requested from the corresponding author.

## Abbreviations

*CI*: Confidence interval
DOH: Department of Health
MAT: Microscopic agglutination test
NPV: Negative predictive value
PCR: Polymerase chain reaction
PPV: Positive predictive value
qPCR: Real-time polymerase chain reaction
