# Supplementary material for: Evaluating the diagnostic accuracy of clinical judgement and rapid tests for leptospirosis in the Philippines: implications for public health management
Source: Infect Dis Poverty. 2026 Jan 22;15:14. doi: 10.1186/s40249-026-01413-0 (PMC12825197; doi:10.1186/s40249-026-01413-0)
Supplement: Supplementary file 1 — Supplementary material 1. [file 40249_2026_1413_MOESM1_ESM.docx]

**Supplemental Information**

| Supplemental Table 1. Comparison of physicians’ clinical judgement with the results of institutions’ laboratory rapid testing in Hospital A using the Acon^®^ Leptospira IgG/IgM Rapid Test, Acon Laboratories, USA (*n* = 13) | | | | | | | | |
| --- | --- | --- | --- | --- | --- | --- | --- | --- |
|  | | MAT | | Sensitivity (95% *CI*) | Specificity (95% *CI*) | PPV  (95% *CI*) | NPV  (95% *CI*) | Accuracy (95% *CI*) |
|  |  | Positive | Negative |  |  |  |  |  |
| Clinical Judgement | Positive | 4 | 0 | 44.4% (13.7%–78.8%) | 100.0% (39.8%–100.0%) | 100.0% (39.8%–100.0%) | 44.4% (30.8%–58.9%) | 61.5% (31.6%–86.1%) |
|  | Negative | 5 | 4 |  |  |  |  |  |
| Laboratory Rapid Test Kit | Positive | 3 | 0 | 33.3% (7.5%–70.1%) | 100.0% (39.8%–100.0%) | 100.0% (29.4%–100.0%) | 40.0% (29.6%–51.4%) | 53.9% (25.1%–80.8%) |
|  | Negative | 6 | 4 |  |  |  |  |  |
| *CI*, confidence interval; PPV, positive predictive value; NPV, negative predictive value; MAT, microscopic agglutination test. | | | | | | | | |

| Supplemental Table 2. Comparison of physicians’ clinical judgement with the results of institutions’ laboratory rapid testing in Hospital B using the Aria^®^ Leptospira IgG/IgM Combo Rapid Test, CTK Biotech, USA (*n* = 59) | | | | | | | | |
| --- | --- | --- | --- | --- | --- | --- | --- | --- |
|  | | MAT | | Sensitivity (95% *CI*) | Specificity (95% *CI*) | PPV  (95% *CI*) | NPV  (95% *CI*) | Accuracy (95% *CI*) |
|  |  | Positive | Negative |  |  |  |  |  |
| Clinical Judgement | Positive | 32 | 10 | 76.2% (60.6%–88.0%) | 41.2% (18.4%–67.1%) | 76.2% (67.5%–83.1%) | 41.2% (24.2%–60.5%) | 66.1% (52.6%–77.9%) |
|  | Negative | 10 | 7 |  |  |  |  |  |
| Laboratory Rapid Test Kit | Positive | 20 | 3 | 46.5% (31.2%–62.4%) | 81.3% (54.4%–96.0%) | 87.0% (69.6%–95.1%) | 36.1% (28.2%–44.9%) | 55.9% (42.4%–68.8%) |
|  | Negative | 23 | 13 |  |  |  |  |  |
| *CI*, confidence interval; PPV, positive predictive value; NPV, negative predictive value; MAT, microscopic agglutination test. | | | | | | | | |

| Supplemental Table 3. Comparison of physicians’ clinical judgement with the results of institutions’ laboratory rapid testing in Hospital C using the Basecheck^TM^ Leptospira IgG/IgM Rapid Test Cassette, Acro Biotech, China (*n* = 55) | | | | | | | | |
| --- | --- | --- | --- | --- | --- | --- | --- | --- |
|  | | MAT | | Sensitivity (95% *CI*) | Specificity (95% *CI*) | PPV  (95% *CI*) | NPV  (95% *CI*) | Accuracy (95% *CI*) |
|  |  | Positive | Negative |  |  |  |  |  |
| Clinical Judgement | Positive | 21 | 25 | 87.5% (67.6%–97.3%) | 19.4% (7.5%–37.5%) | 45.7% (40.0%–51.4%) | 66.7% (35.8%–87.8%) | 49.1% (35.4%–62.9%) |
|  | Negative | 3 | 6 |  |  |  |  |  |
| Laboratory Rapid Test Kit | Positive | 9 | 6 | 39.1% (19.7%–61.5%) | 81.3% (63.6%–92.8%) | 60.0% (38.3%–78.4%) | 65.0% (56.3%–72.8%) | 63.6% (49.6%–76.2%) |
|  | Negative | 14 | 26 |  |  |  |  |  |
| *CI*, confidence interval; PPV, positive predictive value; NPV, negative predictive value; MAT, microscopic agglutination test. | | | | | | | | |
